# Development and pilot testing of a health education program to improve immigrants’ access to Canadian health services

**DOI:** 10.1186/s12913-020-05180-y

**Published:** 2020-04-17

**Authors:** Setareh Ghahari, Shawna Burnett, Libby Alexander

**Affiliations:** School of Rehabilitation Therapy, Queen’s University, Kingston, Ontario Canada

**Keywords:** Canada, Immigrants, Health education, Healthcare access

## Abstract

**Background:**

In Canada’s increasing immigrant population, a phenomenon called the “healthy immigrant effect” has arisen in which health declines after four years of settling. Access to healthcare is an important consideration. There is strong evidence that immigrants lack confidence and knowledge for navigating health services. The aim of this study was to develop and pilot test the Accessing Canadian Healthcare for Immigrants: Empowerment, Voice & Enablement (ACHIEVE) program.

**Method:**

The study employed an exploratory sequential mixed methods design. A qualitative study was completed. Program content was developed based on a scoping review and refined in a formative evaluation. Then, a pilot test of the program measured participants’ perceived efficacy in improving confidence in healthcare navigation, program satisfaction, and learning in individual sessions.

**Results:**

Researchers found significantly higher rates of health navigation and an increase in knowledge about the Canadian health system post-program.

**Conclusions:**

Results provide promising evidence that ACHIEVE may improve confidence in healthcare access among immigrants, demonstrating potential for dispersion on a larger scale.

## New contribution to the literature

Immigrants are a vulnerable population that require special attention with regards to improving their Canadian health literacy. The ACHIEVE program has demonstrated promising results in providing health education to immigrants. Further study is required to determine the program’s effectiveness on a larger scale.

## Background

Canada, being one of the main immigration destinations in the world, is planning to welcome 350,000 new immigrants in 2021 [1]. Immigrants typically arrive with above-average health [2], a well-known phenomenon called the “healthy immigrant effect”. However, 4 years after settling, immigrants’ health shows a significant decline [3–5]. Alcohol consumption [6], obesity [2], depression [6–8] and chronic disease [2, 5, 9, 10] typically increase to levels significantly higher than in the native-born population [11].

This change in health status years after immigrants settle into a country is not only seen in Canada. Other common destinations for immigrants i.e. US and Australia, are facing a similar problem [11]. In European countries, among the minority that have policies specific to immigrant health, there is a divergent array targeting specific health issues as well as supply (provider) and demand (patient) aspects [12]. Supply side policies include training to promote equality in healthcare, through such concepts as intercultural competence, as well as organizational change to improve services. Demand-side interventions include promoting care-seeking behaviour and improving health education and patient-provider communication.

In Canada, health care is organized provincially and must meet the standards of the Canada Health Act which include: public administration, comprehensiveness, universality, portability and accessibility [13]. Local settlement services such as the London Cross Cultural Learner Centre (http://www.lcclc.org/) [14] and Immigrant Services Kingston and Area (https://kchc.ca/weller-avenue/immigrant-services/) [15] can provide newcomers with information on healthcare, including how to apply for provincial coverage. New immigrants in Canada must wait at least 3 months before receiving coverage. During this period, they are expected apply for temporary private coverage or access health care through community health centres for lower cost services [16]. In Ontario, the government provides information on how to find a primary care physician, which can be difficult in some parts of the province. New immigrants are advised to use walk-in clinics while they are without a general practitioner. Telemedicine is another service where people can obtain health advice from registered nurses in English and French, as well as translation for some languages [16].

In order to have a healthy population, access to health services is essential. Healthcare access can be defined as the ability to navigate health services and communicate effectively with healthcare providers [17]. But immigrants do not have adequate access to health services. A scoping review completed by this research team has revealed that accessing the Canadian healthcare (CHC) system has been shown to be a monumental obstacle course to immigrants nationwide and immigrants experience several barriers in accessing the services [18]. The barriers are characterized by a lack of confidence and self-advocacy as well as cultural differences. Low confidence and poor language proficiency have been shown to decrease self-advocacy in navigating Canadian healthcare system in immigrants and prevent them from effectively expressing their health needs [18]. Cultural differences form another barrier to accessing healthcare services because they can shape various health behaviours through beliefs such as not wanting to feel like a burden or dismissing the relevance of a medical procedure in one’s life [18].

In Canada, lack of information on how to access or navigate services is also a common barrier experienced by immigrants [18]. In terms of service utilization in Canada, some research has found little difference between immigrants and Canadian-born individuals [9]. However Tiagi’s [19] analysis shows that recent immigrants are more likely to visit the emergency room and less likely to see a general practitioner compared to native-born Canadians. This can compromise efficiency in emergency services and continuity of care for newcomers [19]. Possible explanations can include the lack of available general practitioners, negative perceptions of Canadian health care [20] and communication difficulties [21].

While there are information resources for newcomers [16, 22], as Fernàndez-Gutiérez and colleagues [23] note it is important to promote *interactive* and *critical* [24] health literacy: skills that enable immigrants to actively and critically navigate this information. To overcome these barriers, the literature suggests education programs for immigrants to enable timely access to health services and effective navigation of the healthcare system [18, 25]. A review on health literacy interventions for immigrant populations found few specific programs for immigrant populations and those that existed only addressed disease-specific areas of health literacy such as HIV/AIDS, various forms of cancer, diabetes, and cardiovascular disease [23]. None of the current educational programs address general understanding of health services and confidence in navigating the system.

Therefore, the aims of the study were to develop an evidence-based health education program to address barriers that immigrants may face in accessing healthcare services in Canada; and to pilot test the program in a sample of immigrants. This study was approved for ethics at the Queen’s University Ethics Review Board.

## Methods

This paper describes the development and piloting testing of a healthcare access program named as ‘Accessing Canadian Healthcare for Immigrants: Empowerment, Voice, and Enablement’ (ACHIEVE). An exploratory sequential mixed methods design was employed to address the objectives of the study [26].

First we conducted a qualitative study (Study 1) to identify areas of health literacy missing for Canadian immigrants that would be important for ACHIEVE’s content.

Second, we developed the program content (Study 2) in two steps.

Step 1: Based on a previously conducted scoping review by the research team [18] and the results of the qualitative study, we developed the preliminary content of the program.

Step 2: The content of the program was refined in a formative evaluation through several rounds of consultation with stakeholders/experts to refine the content.

Finally we conducted a quantitative pilot study (Study 3) to assess program effectiveness in improving confidence in accessing healthcare services.

Due to the variable nature of the immigrant population, in this paper, we will reference immigrants as individuals who had moved from their home country to another country, regardless of year of landing and class of entry [27] and we use the term refugee to reference a sub group of immigrants who are driven from their homes and communities due to fear of persecution and are forced to seek protection in another country [28].

### Study 1: qualitative study to explore the barriers to healthcare faced by immigrants

A qualitative study was employed, specifically with an interview process in order to obtain locally pertinent narratives [29] It was completed to explore the barriers, beyond language, that immigrants face when accessing the Canadian health care system. Both immigrants and healthcare providers were recruited for this study. Maximum variation sampling through snowball sampling [30] was used for both groups. To recruit participants, individuals from the researchers’ social networks with diverse countries of origin were contacted through email. A study flyer was also distributed through Immigrant Services Kingston and Area. Those who were interested in the study received an information sheet and the interview questions in order to make an informed decision to participate in the study. Referrals from the initial participants were then used to recruit additional participants for the study.

Immigrants were included if they were 18 years or older, had experience accessing the Canadian health care system, and expressed interest in participating. Participants were required to be fluent in English, meaning that they were able to effectively communicate in English to work and/or study in Canada. Immigrant participants who met the inclusion criteria and whose experiences were predicted to allow for information to be collected from diverse perspectives (maximum variation) were included in the study based on their ethnicity, home country, years of life in Canada, and type of immigration.

Healthcare providers were included in the study if they were working in primary care (family doctors, nurses, and specialists) and had extensive clinical experience working with the immigrant population in the Canadian health care system (at least for 2 years). Out of the six healthcare providers who were recruited to the study, two were native to Canada and the other four were intentionally chosen to be immigrants.

Interviews were conducted by occupational therapy students and were on average 45 min. Interviews from both the immigrant participants and healthcare providers were transcribed verbatim and all data was analyzed together. Data analysis was conducted by an occupational therapist under the supervision of the first author, who is also an occupational therapist and has experience with navigating the Canadian healthcare system as a newcomer. Analysis consisted of immersion in the data, substantive coding, comparing, and data sorting [31]. The transcripts were read several times to ensure immersion in the data. Substantive coding was conducted, during which, the data was examined line by line. Each piece of relevant data was given a detailed descriptive code. The codes from different interviews were systematically compared and contrasted, which resulted in complex and inclusive categories. The same codes and categories were used for both the immigrant and HCP interviews. The categories were then sorted through the process of card sorting [32], which involves combining common codes together to create categories. The card sorting results were independently reviewed by two other research team members to ensure accurate and objective categorization of codes.

### Study 2: Development of program content

The development of program content was completed in two steps.

#### Step 1: Development of preliminary content

Based on the scoping review previously conducted by the research team [18] and qualitative study results, the ACHIEVE program was designed in collaboration with researchers, community organizations, and immigrants, thus blending both theoretical and experiential knowledge. The program development involved Loyola School of Adult and Continuing Education, Kingston Employment for Youth (KEYS) Job Centre, and Immigrant Services Kingston and Area. The combination of community organizations and immigrant experts reviewed the content and also suggested the format and method of delivery that best suits immigrant cultural needs. An additional expert consultation with immigrants and settlement workers for information about immigrants’ experience and information needs helped modify the content of the program. The Canadian Ministry of Health website (https://www.ontario.ca/page/ministry-health) aided in providing specific information on the Canadian healthcare system.

Further, content as well as the flow and order of information was reviewed by three English as a Second Language (ESL) teachers ensured appropriate English literacy level (Canadian Language Benchmark Level 3) [33]. In addition, we learned from immigrants in these consultations that they refer to their ESL teachers as a resource for finding answers to many of their questions including health-related questions. Therefore, ESL teachers also were asked to add/remove/revise the content based on their experience with health-related questions they receive from their students. After each expert review, the content was revised before the next expert was invited to review the next version.

#### Step 2: Formative evaluation

During formative evaluation of the program, we refined the ACHIEVE content in two ESL classes (total *n* = 20) in which verbal feedback was received from both ESL teachers and newcomers. The facilitators of the ACHIEVE program were members of the research team who had first-hand experience as Canadian immigrants as well as had a large involvement in ACHIEVE content creation. An observer who was familiar with the content but was not involve in the content development, took notes during those sessions on timing, as well as questions and issues that arose. After delivery of each class, the program content was revised.

Upon completion of the above two steps, the research team finalized further development of the program through implementing the suggestions brought forth as well as revisiting the literature on immigrants’ health needs. The program aimed to assist immigrants’ healthcare access by improving their knowledge, skills and confidence accessing the Canadian healthcare system. To address these needs, the goals of the program are to enable immigrants to build:

- better healthcare communication skills and self-advocacy
- knowledge of available resources
- self-efficacy in finding resources in the community and social service systems, and
- improved community integration of immigrants.

### Study 3. Quantitative pilot study

In the pilot pre-post study, we hypothesized that immigrants who receive the ACHIEVE program would report better communication with healthcare providers and have improved confidence in navigating services compared to their baseline.

### Participants

All immigrants who showed interest in the program participated in ACHIEVE. However, data were collected only from those who provided written consent to participate in the study. Immigrants were included if they were adults (over age 18) and had an English level of 3 or above which, according to Canadian Language Benchmark standards (CLB) standards, indicates the ability to comprehend short and simple conversation [33].

Immigrant participants were recruited in Kingston, Ontario through two organizations: KEYS Job Centre and Loyola School of Adult and Continuing Education. KEYS offers specialized services to immigrants, including a mentoring program, refugee resettlement services ESL classes. The Loyola School supports the return of former students to the education system and also offers ESL classes. Students who were already attending classes, which corresponded to different CLB proficiency levels, were recruited for the study.

### Measures

Demographic information was collected once at the beginning of the program to record participant age, type of immigration, family status, and years lived in Canada. Two questionnaires were also used to test the efficacy of the program through participants’ understanding and confidence in healthcare access.

1. The Health Education Impact Questionnaire (heiQ) by Osborne et al. [34], measures self-reported understanding and confidence in communicative ability with health organizations and health professionals. The Health Navigation Scale measured participants’ self-perceived confidence in navigating health services both before and after the entire ACHIEVE program. This scale consists of 5 items on a 4-point Likert scale ranging from 1 (strongly disagree) to 4 (strongly agree) with a Cronbach’s alpha of .82 [34] The Program Evaluation Scale that is a component of the heiQ was conducted solely after program completion to measure satisfaction with the program overall in successfully teaching health literacy. This scale consists of 9 items on a 4-point Likert scale ranging from 1 (strongly disagree) to 4 (strongly agree), with a maximum score of 36. Permission to use the tool was obtained from the authors.
2. The second questionnaire entitled “Confidence in Health Access” was created by the research team for this study after an exhaustive literature review and consultation by immigrant experts. The questionnaires are available as an additional file in this manuscript. Questions are based on the ACHIEVE program content with 2–3 questions ranked 0–5 (maximum scores on questionnaires therefore ranging from 10 to 15). Questionnaires were administered at the beginning and end of each weekly session to assess comprehension of course concepts. Face validity of the questionnaire was evaluated by review of the measure by ESL teachers and settlement workers who were familiar with the content of the ACHIEVE program. All reviewers agreed that the questions do measure understanding and confidence that is expected after participation in the program. Their suggestions for simplifying the wording of the questions were implemented before start of the program.
3. Four open ended questions were included in the questionnaire to collect the participants’ general feedback on the participants about what they liked most about the program, what they liked least about the program, what they would add and what they would change from the program.

### Analysis

A paired samples *t*-test using Statistical Package for Social Sciences (SPSS) was calculated to test the effectiveness of the program in improving confidence in accessing Canadian healthcare (*p* < .05). The heiQ questionnaire was used to measure participant satisfaction in the program using the Program Evaluation Scale. As the data violated assumptions of normality, Wilcoxon Signed Rank tests were calculated to determine the effectiveness of each individual session. The results from the three open ended questions were tabulated.

## Results

### Study 1: Qualitative study to explore the barriers to healthcare faced by immigrants

This study included 11 immigrants (8 females and 3 males) and 6 HCPs (4 females and 2 males). Immigrant participants originated from a diverse range of countries and varied in the time since immigration. These participants also had a range of employment and marital statuses. All immigrant participants had post-secondary education ranging from trade/vocation school, master’s or doctoral degree, and professional designations. Immigrant participants spoke of accessing the Canadian health care system at different time points depending on their needs at each stage of life. For example, some participants reported medical events such as labour and the birthing process as well as attending the emergency room (ER) after an accident.

The HCPs included a family doctor (*n* = 1), physician specialists (*n* = 4), and a registered nurse (*n* = 1), each with a wide range of clinical experience. Of the six HCPs, two were native to Canada and the other four were intentionally chosen as they themselves were immigrants. Time since immigration to Canada for the HCPs ranged from 10 to 28 years.

Through data analysis, barriers to access were described at the individual, community, and system levels. Under each category, several subcategories of barriers were identified. Table 1 includes quotations from participants discussing identified barriers.

**Table 1:** Examples of Identified Barriers to Healthcare by Participants

Barrier	Quote
Language and Communication (Terminology describing sickness)	When I was finally seen by a doctor, we talked, and he tried to get the story of what happened but at the time my English was not that good so I struggled to communicate what exactly had happened. So by the time I finally pieced my way into explaining what had happened it had already been another 20 to 30 min and by that time he didn’t have a lot of time for treatment left (Participant 23).
Adopting the Canadian System (Restricted Medication access)	I find it so frustrating, cause’ I already know what [is wrong with me] and I know what I need, so I already don’t feel good, but I still have to go to the office … and wait there … and then get prescription and go to the pharmacy (Participant 12).
Adopting the Canadian System (Differentiating between services)	In [my home country] you don’t go to the hospital for anything unless you are dying and have a surgery. So here, having to go to the hospital every time that my son had a cough or a fever or a …, you know, it was a real shock for me and I hated it (Participant 11). At first it was just a different system from the one we are used to back home. Just the concept of a walk-in clinic being different from a family doctors office. That was just a weird thing to get used to (Participant 21).
Adopting the Canadian System (Perceived Shortage of Doctors)	My impression is that, there is not enough doctors, not enough services … that you can access. For instance, in [my home country], there’s maybe too many doctors and too many services. And there is doctor offices at every corner of the street (Participant 12).
Adopting the Canadian System (Limited Options)	[In my home country] it’s so easy to say when you are getting a diagnosis, like ‘oh, I will just go get a second opinion’, right? And I used to have that, but here where do you go to get a second opinion? You have one family doctor that you … took you so long to find who you might not know how to convince to think about other options (Participant 12).
Transportation (Language and Communication)	[When I first came to Canada] people would tell us get on [that bus] and even then it was hard figuring out where to get off. Like, street names, you can read them but then it gets pronounced by someone and you’re looking out the window trying to see if you just past that (Participant 24).
Transportation (Limited Finances)	The nearest medical centre, in terms of walking distance, was about a half an hour walk. And there were times where either my sister or I would be too sick to make the walk so our parents would have to carry us all that distance, so it definitely wasn’t easy getting there (Participant 23).
Transportation (Understanding Transit systems)	Transportation in general was tough, in terms of figuring out just how much you’re paying for bus tickets, like, getting transfers. In Ottawa, there are the Gatineau buses and the Ottawa buses, so like just figuring out which bus to get on, and there’s obviously like a billion buses … (Participant 24)
Unfamiliar with Available Services	[What is missing is] where do they start? Like where is the place for immigrants that you could go to and they could tell you, ‘well, if you actually need a dentist, or if you need a hairdresser, or if you need an optometrist, like these are the most popular, or these are the good ones in the city, or …’ you know? (Participant 11)
Limited Access to Family Doctors and Specialists	Sometime if I don’t get an appointment with a family doctor I just go to the walk in, you know what I need I have to wait probably 1 h max but still you know. Not like when you’re setting an appointment with the family doctor you have to wait for 2 to 3 days and like ok my fever will be gone by then. (Participant 12).
Limited Access to Family Doctors and Specialists	We had the probation period to wait and if your little one gets sick then that can get quite worrying, like if it is a very bad, you know like some kind of bad flu or something really even more threatening than that would be my concern. We did not know where to go and get help (Participant 10).

*Individual level barriers*, defined as those that arise when individuals interact with each other on a person-to-person basis, included: language and communication, lack of cultural competency among HCPs and adapting to the Canadian health care system. This has a significant effect on community integration of immigrants. Key skills that have been shown to be missing include lack of knowledge and skills to navigate social systems; lack of understanding about how the system works; and lack of familiarity and self-efficacy in accessing available resources.

*Community level barriers* are those that prevent a person from utilizing the resources available in his or her community. These barriers arise because of a lack of resources, a lack of understanding of how to use these resources, or a lack of physical access to the needed resources. Such barriers included transportation and being unfamiliar with available resources and services.

Finally, *system level barriers* are defined as those that prevent a person from interacting with a system that is larger than their immediate community. These included limited access to family doctors and specialists and limited access to health benefits.

### Study 2: Development of program content

#### Step 1: Development of preliminary content

Based on the results of our team’s previously published scoping review and the qualitative study, the following topics were determined to be included in the program:

1. Introduction to the Ontario healthcare system
2. Information on family doctors
3. Healthcare and symptoms communication
4. Overcoming specific barriers such as preventing illness and cultural differences

The content was prepared with help from Ministry of Health website information to follow the Ontario healthcare system (https://www.ontario.ca/page/ministry-health) and the flow and order of information were reviewed by expert consultation including immigrants and settlement workers. The content was next reviewed by ESL teachers to ensure appropriate English literacy level for those immigrants with Canadian Language Benchmark Standards of Level 3 or higher.

The initial formative evaluation, using ESL classes (*n* = 20), showed that the 4 subject areas provide a general overview of the CHC, however comments of the program by immigrants showed participants were interested in hearing about specific areas of health literacy in addition to CHC overview. In an additional literary review on specific needs, skills and knowledge on accessing mental health services were identified as there is a presence of depression and anxiety among many immigrants and refugees—often due to migration stress and adjustment issues [35–37]. Severely low rates of accessing long term care for mental health have emerged due to disengagement with health services, as well as both pre- and post-migration negatively affecting mental health [38]. To combat this serious issue, the research team chose to dedicate two additional sessions to mental health. Furthermore, lack of information related to sexual health in some cases led to misconceptions about healthcare [39, 40]. Therefore, the addition of mental and sexual health sessions in ACHIEVE ensured that immigrants are educated on the specialized areas of health literacy that are in demand for this population.

Consequently, the following subjects were also added to the program:

5. Introduction to mental health
6. Where and how to get help for mental health
7. Where and how to get help for sexual health

A detailed description of each ACHIEVE session is presented in Table 2.

**Table 2:** ACHIEVE Program Sessions

Session Number and Title	Details
Session 1: The Ontario Healthcare System	• How to obtain and renew a health card • Ontario Health Insurance Plan coverage • Emergency services
Session 2: Family Doctors	• Finding a family doctor • Specialist services • Confidentiality
Session 3: Communication	• Common symptoms (headache, fever) • How to book an appointment • Accessing valid health information
Session 4: Overcoming Specific Barriers	• Vaccinations • Screening tests • Lyme disease
Session 5: Mental Health I	• Common mental illness in immigrant populations • Good and poor mental health • Stigmatization of mental illness
Session 6: Mental Health II	• Types of doctors in mental health • Wellness exercises
Session 7: Sexual Health	• Types of contraceptives • Sexually Transmitted Infection (STI) prevention • Maternal health

The complete ACHIEVE curriculum

The final version of ACHIEVE runs in a classroom environment with one 2.5 h session per week. Sessions include lectures, group discussions, class activities, and homework assignments. Participants are provided with study documents to help them follow along with session content. Program facilitators were newer members of the research team who had been trained during a half day training session. They received a Facilitator’s Manual that included details about each topic, answers to possible questions from immigrants, and tips for how to run a group program based on the principle of chronic disease self-management [41], brief action planning (BAP) [42], and self-efficacy theory [43].

### Study 3. Quantitative pilot study

In total, 46 participants gave consent to participate in our research. Of these participants, 21 were females, 21 were male, and 4 had missing data for gender. For more in-depth age and other demographic frequencies, see Table 3. The average consenting number of participants in each session was 21.

**Table 3:** Participant Demographics

Variable	Number of Participants n (%)
**Age Group**	
20–30	10 (21.7)
31–40	13 (28.3)
41–50	10 (21.7)
51–74	6 (13)
Missing	7 (15.2)
**Type of Immigration**	
Family	14 (30.4)
Refugee	16 (34.8)
Skilled worker	2 (4.3)
Prefer not to answer	5 (10.9)
Missing	9 (19.6)
**Family Status: Number of Children**	
No children	4 (8.7)
1–2	13 (28.3)
4–6	7 (15.2)
Missing	22 (47.8)
**Family Status: Martial Status**	
Married/Common-law	29 (63.0)
Single	8 (17.4)
Divorced/Separated	3 (6.5)
Widowed	1 (2.2)
Prefer not to answer	1 (2.2)
Missing	4 (8.7)
**Year Landed in Canada (Most Popular)**	
2016	7 (15.2)
2017	16 (34.8)
2018	4 (8.7)
**Birth Country (Most Popular)**	
Syria	13 (28.3)
China	7 (15.2)
Burundi	3 (6.5)

Participant demographics in frequencies

### Overall health navigation and program evaluation

Of the 46 consenting participants, 20 completed both the pre-test and the post-test heiQ. The primary analysis of pre-post heiQ Health Navigation Scale scores met all assumptions including normality. Scores were summed and a paired samples t-test was conducted to compare health navigation before and after the 7-week long ACHIEVE program. The test demonstrated that post-test scores (M = 17.00) were significantly higher than pre-test scores (M = 14.20, t (19) = − 3.91, *p* = 0.001).

Self-report measures indicating participants’ perceptions of the ACHIEVE program were completed by 22 participants using the heiQ Program Evaluation Scale, with a potential maximum score of 36. As this scale measured participants’ satisfaction in the program, it was only distributed post-session. Descriptive statistics were as follows, M = 32.45, Min = 24.00, Max = 36.00, SD = 3.83.

### Weekly comprehension

**Fig. 1:**
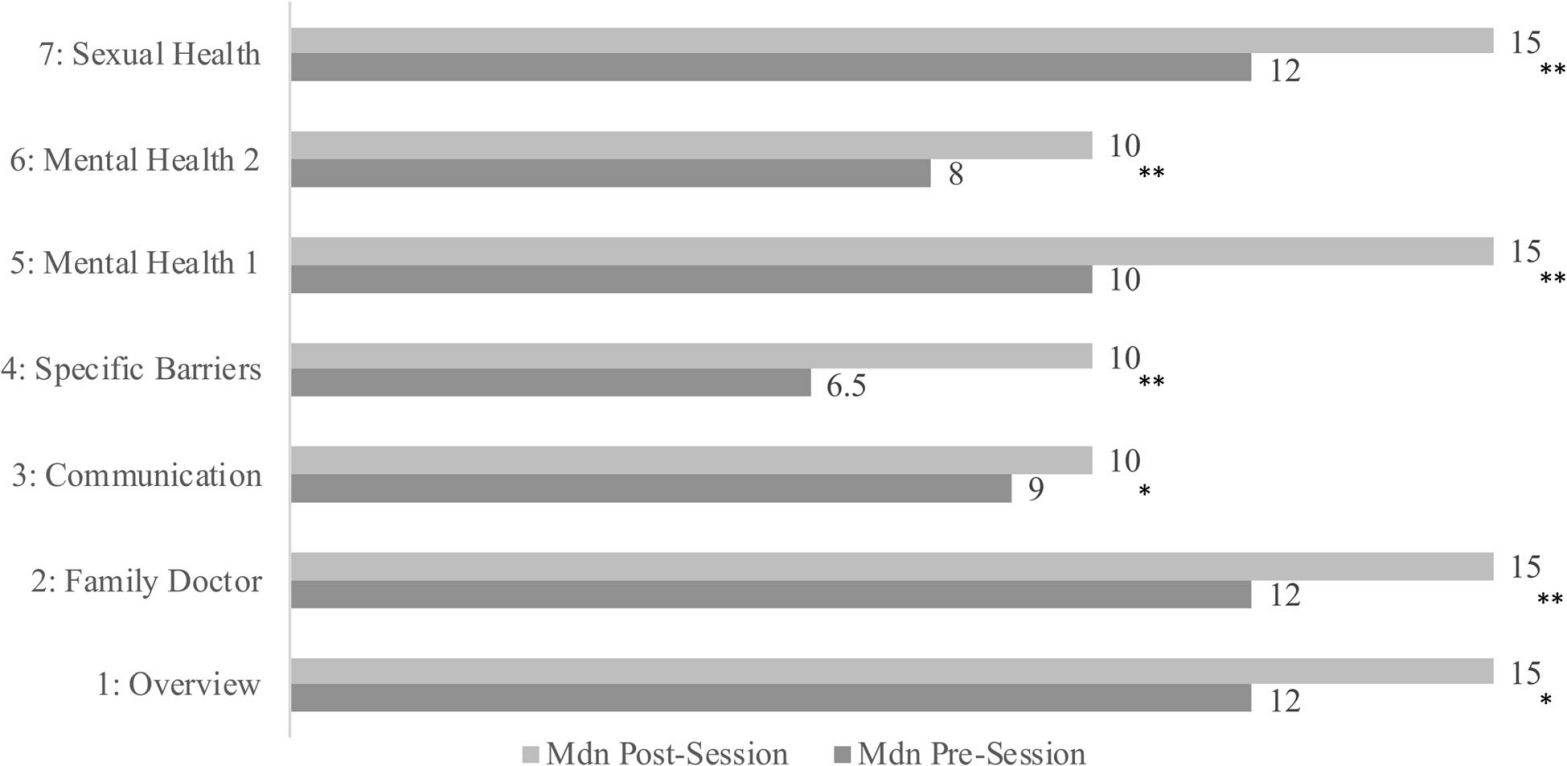
Weekly Learning. Depicts the pre- and post- weekly session scores on program content that demonstrates learning in individual sessions

The analysis of weekly comprehension of course concepts using our team’s Confidence in Health Access Questionnaires violated assumptions of normality therefore, medians and Wilcoxon Signed Rank tests were calculated. Pre- and post- test descriptive statistics as well as significance are displayed in Fig. 1.

The open-ended questions provided noteworthy self-reports of participants’ perception of the program. When asked what could be changed or what they liked the least in the ACHIEVE program, all participants reported nothing or did not answer the question. When asked what they liked the most, the majority of participants named the mental health sessions. Reports on what could be added to sessions included family health, information on different types of cancers, and nutrition.

Overall Results.

A complete table of results for the two heiQ scales on Health Navigation and Program Evaluation, in addition to the seven Confidence in Access Questionnaires are available in Table 4.

**Table 4:** Complete ACHIEVE Results

Variable	Median Pre-Test	Median Post-Test	Maximum Possible Score	Significance
Health Education Impact Questionnaire (heiQ): Health Navigation Scale				
	M = 14.20	M = 17.00	Max = 20.00	*p* = 0.001
Health Education Impact Questionnaire (heiQ): Program Evaluation Scale				
	N/A	M = 32.45	Max = 36.00	N/A
Confidence in Access Questionnaires				
Session 1: The Ontario Healthcare System	M = 12.00	M = 15.00	Max = 15.00	*p* = 0.020
Session 2: Family Doctors	M = 12.00	M = 15.00	Max = 15.00	*p* = 0.001
Session 3: Communication	M = 9.00	M = 10.00	Max = 10.00	*p* = 0.006
Session 4: Overcoming Specific Barriers	M = 6.50	M = 10.00	Max = 10.00	*p* = 0.000
Session 5: Mental Health I	M = 10.00	M = 15.00	Max = 15.00	*p* = 0.000
Session 6: Mental Health II	M = 8.00	M = 10.00	Max = 10.00	*p* = 0.001
Session 7: Sexual Health	M = 12.00	M = 15.00	Max = 15.00	*p* = 0.000

Complete quantitative results of ACHIEVE pilot program

## Discussion

Low health literacy has been associated with poor use of health care services and substandard health outcomes [44]. Immigrants who are more aware of and have sufficient access to resources are shown to be more resilient when it comes to overcoming healthcare system barriers [45]. By educating Canadian immigrants with an overview of the CHC system, tools for effective communication with healthcare providers, as well as information for accessing health and community resources, immigrants can have tools to overcome barriers to healthcare access negatively affecting their health. Results from the ACHIEVE program found an increase in overall self-reported confidence in health navigation, participant satisfaction, and knowledge. These results show the promise of ACHIEVE in supporting immigrant confidence in accessing the Canadian healthcare system.

It should be noted that the ACHIEVE program, in consultation with stakeholders such as immigrants, settlement workers, and ESL teachers, attempted to make the program as inclusive as possible to prevent barriers to receiving this program’s educational information. For example, much of the program attempts to provide diverse cultural examples in addition to having open discussion around comparing and contrasting Western medicine’s cultural expectations with one’s home country. All immigrants and refugees were encouraged to participate in the program regardless of being undocumented. This was done by ensuring individuals could attend the program even if they did not consent to the team using their information as ESL teacher stakeholders outlined this as an issue for individuals who may be undocumented. Further, demographic information such as type of immigration had an option for “prefer not to answer” and participants were encouraged to share ACHIEVE materials with friends and family for knowledge sharing in the greater population. Facilitators also stayed after class for as long as needed to answer any questions for those with lower English comprehension and ESL teachers would also review content outside of class for those who needed extra time. This extra time was provided in an attempt to ensure adequate health care access and health literacy for all participants.

In revisiting the goals of the program, better healthcare communication skills and self-advocacy was intended to be address in Session 3: *Communication* in which results showed that this session had a significant increase in learning. This session included symptom tables that participants could refer to in the future as well as provided exercises such as making a doctor’s appointment over the phone. It is of interest to note that no participant named this session as the most helpful. Previous health literacy programs have employed ESL teachers directly to run their programs [46, 47] which would likely be the optimal option for teaching immigrants and refugees health literacy as they specialize in educating these populations. However, studies on health programs for immigrants have made attempts to best educate this population. For example, immigrant health programs (including ACHIEVE) have employed the RE-AIM (Reach, Effectiveness, Adoption, Implementation, Maintenance) Framework for health promotion interventions [48] and included stakeholders such as immigrants, ESL teachers, and community personnel to create their program content [49].

For the goal of knowledge of available resources and self-efficacy in finding resources in the community and social service systems, all sessions included an abundance of resources to meet diverse needs with knowledge of multiple cultures in mind. Further, many activities included navigating specific resources and websites to find health information and services. The Confidence in Health Access questionnaire in session 6 had a question regarding resource access that was also found to be statistically significant, (*p* > .01).

Regarding community integration, there was no formal assessment to explore if this goal had been met. However, weekly sessions included discussions amongst the ACHIEVE pilot program participants and facilitators that sparked much discussion around healthcare access and personal experiences. It was observed and reported by facilitators that many participants shared stories surrounding the difficulties of immigrating to a new country, finding a job, and experiencing traumatizing events as refugees— which were all met with peer support that offered a sense of community in the group.

Significant increases in knowledge of CHC topics were found in all weekly sessions showing promising results of potential learning in most sessions. Session 2: *Finding a Family Doctor* showed a large increase in knowledge scores post-session. Previous studies have found that many Canadian immigrants struggle with finding a family doctor, especially undocumented newcomers and those with poor English language abilities [50]. For Session 4: *Overcoming Specific Barriers,* many were not familiar with common CHC tests that were included in the program such as newborn, cervical cancer, and colorectal screening. These findings indicate a need for improved health literacy in accessing preventative healthcare services. Therefore, with the positive results of ACHIEVE’s education on session 4, the program may be effective in implementing such required education.

The additions of sexual and mental health to the ACHIEVE program showed positive quantitative and qualitative results. Sexual health education has been shown to increase knowledge, healthier attitudes, and safe sexual health behaviour [51]. A main point of contact for sexual health education in immigrant youth has been in schools [52] therefore older immigrants aged 18 and above may not have as readily available access to these resources. It may also be beneficial for immigrant parents to understand what their children are learning about sexual health in schools. In accessing mental healthcare among first-generation immigrants, lack of information about services was found to be a barrier [53]. Mental health had the highest increase self-perceived learning in all ACHIEVE sessions which may make the program a good method to combat this barrier.

### Limitations

The limitations provide insight to the considerations for future studies of ACHIEVE as well as when designing future health literacy programs for immigrant and related populations.

Attrition was a significant problem for data collection as many participants would miss classes or leave early for various reasons which prevented us from gathering post-session scores. Of the 46 participants, less than half of the participants (*n* = 20) completed the pre-test and post-test heiQ. In all questionnaires (heiQ, Confidence in Access weekly questionnaires, and demographics), there was missing data. With such significant missing data, there is potential for bias in the final group of respondents as perhaps this only represents those satisfied enough with the program to provide feedback or those with a higher English language ability. There are a variety of reasons for lack of responses including: privacy, questions not perceived to be applicable to participant, and difficulty comprehending the question. This is the nature of ESL classes as research on these programs has demonstrated problems with program completion for a variety of reasons, especially among those with low- to intermediate-level language skills [54].

Secondly, consent and comprehension proved to cause complications among low- to intermediate- level English participants. Earlier trials of ACHIEVE had shown comprehension issues with the consent form and demographic questionnaire, leading the team to remove all “difficult” wording. Even after simplifying the consent form, this document was still observed to be too difficult for some participants to complete. Also, ESL teachers informed the research team that many immigrants, particularly refugees, are hesitant to sign any form of documentation. Further, the potential for undocumented participants or visiting immigrants in the country to participate in ACHIEVE may prevent some from wanting to provide demographic information despite reassurance of confidentiality.

An important limitation to acknowledge is that the “Confidence in Health Access” questionnaire was not validated other than considering the face validity as they had been created by the research team to measure pre- and post- learning for each individual session. It is important to practice caution when interpreting the results as it may not be directly measuring learning.

Given the limitations, the research team may explore shorter sessions to minimize attrition and study designs like wait-list controls to address potential response biases. Future study designs may also explore including a baseline measure a few weeks prior to commencing ACHIEVE to better monitor the effectiveness of the program. Adding a qualitative study to explore the experience of participants was not possible because of their level of English however it should be noted that this would require the use of third parties such as translators in data collection and analysis in order to accurately capture lived experience in participants’ primary language.

## Conclusion

This pilot study has demonstrated positive results for the effectiveness of ACHIEVE in improving confidence in healthcare access and increased knowledge in specific areas of CHC. The research team hopes to replicate the results in a larger sample to find reliable success in the program for nationwide implementation. Future directions for ACHIEVE include creating online modules to improve accessibility of the program. The research team may also customize ACHIEVE to accommodate a range of English proficiency levels.

## Supplementary information

**Additional file 1.** Confidence in Access 1 to 7- short version. Condensed version of Confidence in Access Questionnaires created by the research team. It consists of 7 questionnaires on course content in addition to a pre-program questionnaire and a post-program questionnaire.

**Additional file 2: Figure S1.** Weekly Learning. Depicts the pre- and post- weekly session scores on program content that demonstrates learning in individual sessions.

## Data Availability

The datasets used and/or analysed during the current study are available from the corresponding author on reasonable request.

## Abbreviations

ACHIEVE: Accessing Canadian healthcare for Immigrants: Empowerment, Voice & Enablement
BAP: Brief action planning
CHC: Canadian health care
CLB: Canadian language benchmark
ER: Emergency room
ESL: English as a second language
heiQ: Health education impact questionnaire
KEYS: Kingston Employment Youth Services
RE-AIM: Reach, Effectiveness, Adoption, Implementation, Maintenance
SPSS: Statistical Package for Social Sciences
