# Supplementary material for: Development and pilot testing of a health education program to improve immigrants’ access to Canadian health services
Source: BMC Health Serv Res. 2020 Apr 17;20:321. doi: 10.1186/s12913-020-05180-y (PMC7164356; doi:10.1186/s12913-020-05180-y)
Supplement: Supplementary file 1 — Additional file 1. Confidence in Access 1 to 7- short version. Condensed version of Confidence in Access Questionnaires created by the research team. It consists of 7 questionnaires on course content in addition to a pre-program questionnaire and a post-program questionnaire. [file 12913_2020_5180_MOESM1_ESM.docx]

***Confidence in Health Access***

This ***Confidence in health access*** questionnaire has sentences about your skill or knowledge about accessing health related information or services in Ontario.

Please circle *one* number after each sentence that states what you think.

(At each session, the participants received the questions related to the corresponding content and completed the survey once before and once after completion of the workshop).

| **Sentence** | no  (not ever) | not really | maybe | only a little | yes  (a lot) | not about me |
| --- | --- | --- | --- | --- | --- | --- |
| Week 1 |  |  |  |  |  |  |
| 1. I know what the Ontario Health Insurance Plan (OHIP) is. | 1 | 2 | 3 | 4 | 5 | 0 |
| 1. I feel confident I can keep my OHIP current. | 1 | 2 | 3 | 4 | 5 | 0 |
| 1. I feel confident using the emergency room if needed. | 1 | 2 | 3 | 4 | 5 | 0 |
| Week 2 |  |  |  |  |  |  |
| 1. I know my rights as a patient in Canada. | 1 | 2 | 3 | 4 | 5 | 0 |
| 1. I feel confident that I can question others if they do not keep my health information private. | 1 | 2 | 3 | 4 | 5 | 0 |
| 1. I know why it is important for me to have a family doctor. | 1 | 2 | 3 | 4 | 5 | 0 |
| Week 3 |  |  |  |  |  |  |
| 1. I feel confident talking about my cultural needs with healthcare professionals. | 1 | 2 | 3 | 4 | 5 | 0 |
| 1. I feel confident in my ability to explain what is wrong or talk about my health. | 1 | 2 | 3 | 4 | 5 | 0 |
| Week 4 |  |  |  |  |  |  |
| 1. I feel confident in my ability to get any health care information that I may need | 1 | 2 | 3 | 4 | 5 | 0 |
| 1. I know what screening tests are available | 1 | 2 | 3 | 4 | 5 | 0 |
| Week 5 |  |  |  |  |  |  |
| 1. I know what mental health means. | 1 | 2 | 3 | 4 | 5 | 0 |
| 1. I know some symptoms of mental illnesses. | 1 | 2 | 3 | 4 | 5 | 0 |
| 1. I know when to refer (or refer my family members) to health professionals to get help for mental health problems | 1 | 2 | 3 | 4 | 5 | 0 |
| Week 6 |  |  |  |  |  |  |
| 1. I understand why we need to stop the negative feelings against people with mental illness | 1 | 2 | 3 | 4 | 5 | 0 |
| 1. I know some symptoms of mental illnesses and I feel confident in getting the right resources for help with my mental health if I needed it | 1 | 2 | 3 | 4 | 5 | 0 |
| Week 7 |  |  |  |  |  |  |
| 1. I know how birth control works. | 1 | 2 | 3 | 4 | 5 | 0 |
| 1. I feel confident protecting myself during sexual activities. | 1 | 2 | 3 | 4 | 5 | 0 |
| 1. I feel confident knowing where to go for help if I am (or my partner is) pregnant. | 1 | 2 | 3 | 4 | 5 | 0 |
