# Supplementary material for: Development and pilot testing of a health education program to improve immigrants’ access to Canadian health services
Source: BMC Health Serv Res. 2020 Apr 17;20:321. doi: 10.1186/s12913-020-05180-y (PMC7164356; doi:10.1186/s12913-020-05180-y)
Supplement: Supplementary file 2 — Additional file 2: Figure S1. Weekly Learning. Depicts the pre- and post- weekly session scores on program content that demonstrates learning in individual sessions. [file 12913_2020_5180_MOESM2_ESM.docx]

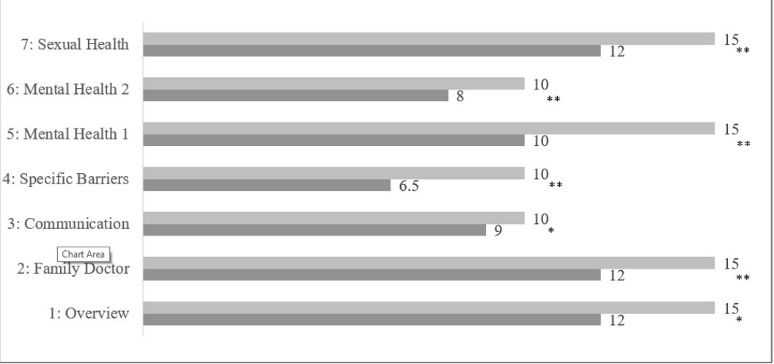


*Descriptive statistics and significance of participants’ self-perceived pre- and post-session knowledge in weekly sessions.* Represent a significant increase in self-perceived knowledge post-session (* p < or = .05; ** p < or = .001).*
